# Competency Level in Generation and Usage of Health Information Within the Landscape of Ghana

**DOI:** 10.1155/bmri/8826168

**Published:** 2025-09-10

**Authors:** Richard Okyere Boadu, Victor Wireko Adu, Kwame Adu Okyere Boadu, Godwin Adzakpah, Nathan Kumasenu Mensah, Emmanuella Esi Arhin, Augustine Ilinkakor Nisanman, Godwin Salakpi, Stephen Ekow Bessabro, Williams Danquah, Idris Adam Simsiah, Emmanuel Obour

**Affiliations:** ^1^ Department of Health Information Management, School of Allied Health Sciences, College of Health and Allied Health Sciences, University of Cape Coast, Cape Coast, Ghana, ucc.edu.gh; ^2^ School of Medicine and Dentistry, College of Health Sciences, Kwame Nkrumah University of Science and Technology, Kumasi, Ghana, knust.edu.gh

**Keywords:** competency, data generation, healthcare professionals, use of health information

## Abstract

**Background:** The ubiquitous nature of data/information in healthcare has made it an imperative facet that requires the services of highly trained professionals with well‐endowed field competencies to properly generate and use this sensitive data to enhance healthcare outcomes. There are still numerous challenges regarding the quality of data generated in the healthcare sector, especially in many middle‐income countries. A growing number of studies show that data quality issues can be linked to the repercussions of inadequate competency levels of some healthcare professionals (HCPs). In that vein, this study was purported to assess the competency level of HCPs regarding the generation and usage of health information.

**Method:** A quantitative cross‐sectional design was employed for the study, where professionals provided self‐ratings of their competencies by completing the structured questionnaire. The study saw a response rate of 98% with 877 HCPs from eight selected health facilities in Ghana. The reliability of the study construct was tested using a Cronbach’s alpha test. The competency level of the professionals was measured on a scale of 1–3 under nine competency areas and categorized into entry, intermediate, and advanced levels. The chi‐square test (*χ* ^2^) and Cramer’s *V* test were used to determine the possibility of any predictive factors associated with the professionals’ competency levels. An ANOVA and a Dunnett’s T3 post hoc test were deployed to ascertain the significant differences in the competency levels attained in the various healthcare facilities involved in the study. All statistical tests resulting in a *p* value less than 0.05 were deemed significant.

**Results:** With a target of 2.30/3.00, HCPs were only found to be mostly competent (advanced level) in the application of health information law and ethics when generating and using health information (2.50) and generic professional skills (2.33). On the contrary, HCPs had low levels of competency in the application of healthcare terminologies and disease classification (1.83), research methods skills (1.94), health service organization and delivery skills (1.96), health information and service organization management skills (2.00), the use of the language of health (2.00), electronic health skills (2.06), and health information records and management skills (2.27). Health information officers and doctors were the only professional categories that attained the threshold in our study. Sex, type of profession, educational level, and years of experience were all identified as significant predictive factors of HCP competency level. There were significant differences in the competency levels of HCPs in various facilities.

**Conclusion:** There are lapses in competency levels about some specific areas which ought to be taken into cognizance. This study concludes that years of experience and educational level are the greatest predictive factors that can affect the competency level of HCPs when it comes to information generation and usage. There is a need for more competency‐based education, capacity building, and in‐service training that will be geared toward the enhancement of HCP competency in the effective generation and usage of data/information to maximize healthcare outcomes.

## 1. Introduction

The conscious effort to improve accountability and ensure evidence‐based decision‐making in healthcare by utilizing limited resources has been a major driver for improving health information (HI) or health‐related data specifically to buttress evidence‐based policy development, planning, management, and evaluation of health services provided [1, 2]. To attain a remarkable level of efficiency in healthcare delivery in various healthcare facilities, data generation and usage should be highly taken into cognizance due to the plethora of merits associated with it [3]. HI or health‐related data is any data that is linked to the physical or mental health of an individual and all the procedures involved in the provision of health services to the individual [4]. It encompasses all types of data related to health status, personal choices regarding treatment selection, health security, and various treatment reports, as well as causes of death and socioeconomic parameters related to health and wellness [5]. It is a collection of information from multiple sources that pertains to human health. It includes data from individual patient records, patient population, clinical and nonclinical data, epidemiological data, demographic data, research data, reference data, and coded data [6, 7].

HI is crucial for the commencement and continuity of care for patients in the healthcare facility. The availability of accurate and timely information to support decision‐making positively affects the quality of healthcare delivery, health planning, and policymaking [8], serving as enough premise to avert possible lapses in health data generated. The voluminous data generated from routine health information systems (RHIS) in the myriad of health facilities ought to be utilized for diverse purposes. Practically, the utilization of the health‐related information/HI is considered to be influenced by three main factors:

i. The attitude and actions of the producers and users of the data generated, which collectively form an integral part of their competency
ii. The technicalities regarding the tools involved in the processing of the data
iii. The organizational context available and supporting the holistic processes involved [8, 9]

The set of knowledge, skills, abilities, and behavior of the healthcare professionals (HCPs) collectively contribute to their individual and organizational performance. Dating back to as early as 2006, Levenson put forward that there is enough evidence to substantiate that competencies are positively related to individual‐level performance and that performance can be improved by mentoring a competency system [10]. On this ground, the competency level of healthcare professionals (CLoHCPs) regarding the generation and usage of HI must not be compromised because it would have an adverse inference on the data generated and the subsequent decisions that will be made. Setting aside the generic competencies in communication, teamwork, problem‐solving, organization and engagement, basic medical knowledge and ethics, which are prerequisites for all HCPs, other specific competencies desirable for quality generation and usage of HI include innumerable scopes or domains that encompass but are not limited to medical knowledge, information technology, information management, information governance, medical classifications and coding, and research methods [11, 12]. For instance, in this modern health sector, just as in any other sector, information communication technologies (ICTs) are rapidly being developed, recommended, and used to improve the quality of work in administration, patient records, health services, and research [13, 14]. In this regard, HCPs are required to be highly trained to be well equipped with the use of the latest information management technology applications [15], to support the fast‐changing need for information in the health sector.

In the attempt to support any organization’s activity, there should be a process design that will suit the overall activities of the organization; data cannot be left out if one wants to accomplish this task. Data is regarded as the most vital asset or primary foundation when it comes to operational, tactical, and decision‐making activities in every organization, including the healthcare sector [16]. Estimates suggest that in 2012, healthcare data reached about 500 petabytes and will keep on expanding to thousands of petabytes [17]. The voluminous data collected during the rendering of services in the healthcare sector are not just generated but analyzed and used for a variety of purposes. They are used for various purposes, including supporting evidence‐based healthcare, continuity of care, billing and reimbursement, public health decision‐making, and national health sector planning [8, 17].

Amid all the technological advancements and other notable information/data‐related interventions inculcated into the health system, studies have stated unequivocally that there are still numerous challenges regarding the quality of data (accuracy, completeness, consistency, and timelessness) generated in the healthcare sector in many low‐ and middle‐income countries (LMICs) [18, 19], which consequently results in the unreliability of information. Poor data quality leads to incorrect decision‐making, and it can mean the difference between life and death when such cases happen in the healthcare sector [20]. On the side of decision‐makers, the reliability of data greatly depends on its quality [20]. The growing number of studies conducted shows that issues of poor data generation, collection, and usage in the health sector can be linked to the repercussions of inadequate competency levels of some HCPs [9, 21, 22].

The prospect of this study is to effectively help mitigate the issues of data quality by ensuring that all health professionals, regardless of their specific discipline, possess a certain level of competencies for operative generation and usage of HI and its system to fulfill an integral part of the WHO building blocks of health systems and World Bank’s “one health system” concept [23, 24]. HCP will be positively imparted to have the aptitude to use and contribute to evidence‐based practices; apply public health informatics in using data, information, and knowledge; assess community health status; develop, evaluate, and implement data‐driven policies, programs, and services; and also engage in professional development [25]. Our study probed and assessed the CLoHCP regarding the generation and usage of HI within the landscape of health and also provided a reliable panacea for what causes the inadequate acquisition of competencies among HCPs about the generation and use of HI or data.

## 2. Research Methods

### 2.1. Study Design

A nonexperimental cross‐sectional quantitative technique was employed for the study to facilitate the collection of data from health professionals who generate and use HI or health‐related data in eight selected health facilities in Ghana. Data collection was done from 1st to 20th May, 2023. The design is purported to help probe and assess the level of competency of HCPs in the selected health facilities regarding the generation and usage of HI or health‐related data and also find out the gaps in these competencies or skills of HCPs.

### 2.2. Study Population

Eight selected health facilities in Ghana were involved in the study, which comprised Suntreso Government Hospital (SGH), Effia Nkwanta Regional Hospital (ENRH), Tarkwa Government Hospital (TGH), University of Cape Coast Hospital (UCCH), Asante‐Akim Central Municipal Hospital (ACMH), Akuse Government Hospital (AGH), Ejura Government Hospital (EGH), and Cape Coast Teaching Hospital (CCTH). The study population included all HCPs in the eight facilities under study who generate and use HI for various purposes. The category of professionals included all health professionals who generate and use HI in various departments and units for various purposes.

### 2.3. Sample Size Determination and Sampling Technique

A total sample size of 898 was determined by using Epi Info statistical software version 7.2.5.0 StatCalc function with a confidence level = 99.9*%*, expected frequency = 50*%*, acceptable margin of error = 0.05 (5%), design effect = 1.0, clusters = 1. The samples were strictly taken only from the population of HCPs who generate or use HI for various reasons by using a simple random sampling technique. This was done to ensure that the main purpose of the research was preserved. The sampling method was used in all the selected facilities under study to prevent bias in the selection of the study participants.

### 2.4. Data Collection Tools and Techniques

A structured questionnaire and a consent form were developed, piloted, and distributed to the targeted study participants as a tool for collecting the needed information from the participants. The researcher distributed the questionnaires himself in two formats, both hardcopy and softcopy, for convenience of the respondents. Nine relevant competency indicators were used as the yardstick or operational definition for measuring the competency levels of the professionals concerning the generation and use of HI. The questionnaire was structured into nine sections for the competency areas of focus. Each of the selected competency areas consisted of some dimensions which facilitated the assessment process. The selected competency areas include generic professional skills (9 dimensions), HI and records management (15 dimensions), language of healthcare (3 dimensions), healthcare terminologies and classification (6 dimensions), research methods (10 dimensions), health services organization and delivery (6 dimensions), HI law and ethics (4 dimensions), eHealth (3 dimensions), and HI services organization and management (19 dimensions) [11]. Participants were allowed to answer the various questions on a Likert scale of 1–3, consisting of entry, intermediate, and advanced levels in respect to their competency under all nine sections.

### 2.5. Measurement and Data Analysis

An assessment was undertaken to ascertain if the participating HCPs possess an adequate level of competencies for the generation and usage of HI. As aforementioned, the ratings of the competency levels were measured on a scale of 1–3. The measured competency levels were designated as follows: 1.0–1.5 (entry), 1.6–2.2 (intermediate), and a targeted level of 2.3–3.0 (advanced). Statistical Package for Social Sciences (SPSS) Version 26 was used to code and transfer the data acquired in the manual format into a digital format for further descriptive and inferential analysis. A Cronbach’s alpha test was used to measure the statistical reliability of the study constructs; the overall Cronbach’s alpha was 0.95. The descriptive data analysis method included means, standard deviation, frequencies, and percentages of the self‐rated score of the HCPs under each of the competency areas of focus. Averages and estimations at a 95% confidence interval for each of the nine competency areas were calculated to represent their overall competency levels. Moreover, further cross‐tabulations and a series of chi‐square (*χ* ^2^) tests of independence were performed on some selected variables to discover any statistically significant association between competency areas and the sociodemographic characteristics of the HCPs. A Cramer’s *V* test was used as a measure of the effect size for association. Again, a one‐way analysis of variance (ANOVA) test with a Dunnett’s T3 post hoc test was performed to find out the actual differences in the overall competency levels of the HCPs across the eight selected facilities. Bartlett’s test was used to ascertain the homogeneity of the variance for the overall score for the facilities. All hypothesis tests resulting in a *p* < 0.05 were considered significant. Finally, visualization techniques were used to create charts to perfectly elaborate on some specific results of the study.

### 2.6. Study Hypotheses

The difference in CLoHCP for generating and using HI was examined across sex, profession type, education level, years of experience, and facilities.

### 2.7. Ethical Considerations

An ethical approval letter (EAL) was obtained from CCTH upon submitting an introductory letter from the Department of Health Information Management, University of Cape Coast, together with detailed research proposal documents. The ethical approval obtained from CCTH is the one that was used to gain permission to undertake the research at the selected healthcare facilities in Ghana. An introductory letter from the Department of Health Information Management, University of Cape Coast, and the EAL obtained from CCTH were submitted to the administrators of all eight facilities for their approval before the data collection procedure began. Moreover, in the process of soliciting responses from the HCPs, their respective informed consents were obtained while the confidentiality of their responses was highly ensured.

## 3. Results

### 3.1. Sociodemographic Characteristics of HCP

The results included the sex, age, educational level, year of experience, and professional type of HCP as their sociodemographic characteristics.

A total of 877 participants, out of the estimated sample size of 898, were successfully recruited for the study, with the detailed distribution indicated in Table 1. The participants were made up of female HCPs representing 59.5% of 877 participants and 40.3% male HCPs, yielding a response rate of 97.7%. Many of the participants fell within the age range of 30–39 years (43.6%). Approximately 4% of them were 50 years old. The participants’ mean age was approximately 33 years. For the professional representation of the participants, the majority, 46.1%, were nurses and midwives. Doctors, nutrition officers, disease control officers, and physician assistants were relatively few at 5.0%, 4.6%, 3.3%, and 3.2%, respectively. In regard to the educational levels of the participants, 44.4% and 41.7% of them held a bachelor’s degree and an HND/diploma, respectively. On account of the years of work experience of the participants, the outcome shows that more than half of the total participants (53.6%) had years of experience of 5 years or less. Notwithstanding that, a considerable number of the participants (26.5%) had 6–10 years of working experience, and 11.7% also had 11–15 years of experience. Participants with 16 years or more of working experience accounted for 8.2%. In aggregation and summary, the mean years of working experience was 6.77 years with an SD of 6.021 within a range of 48. The overall sample distribution among the eight selected health facilities in Ghana that were involved in the study is also indicated in Table 2.

**Table 1:** Sampling distribution among selected health facilities.

**Facility coat**	**No. of participants**	**Percentage (%)**
SGH	125	14.3
AMH	106	12.1
EGH	105	12.0
ENRH	116	13.2
TGH	112	12.8
AGH	94	10.7
CCTH	120	13.6
UCCH	99	11.6
Total	877	100.0

*Note:* Source: survey of healthcare professionals who generate and use health information, 2023.

**Table 2:** Sociodemographic characteristics (*n* = 877).

**Variable**	**Frequency**	**Percentage**
*Sex of respondents*		
Male	353	40.3
Female	522	59.5
No response	2	0.2
*Age group of respondents*		
20–29 years	318	36.3
30–39 years	382	43.6
40–49 years	134	15.3
50 years and above	34	3.9
Unspecified	9	1.0
*Mean = 32.92, SD* ^∗^ * = 10.05, range = 39*		
*Respondent’s profession*		
Doctor ^∗∗∗∗∗∗^	44	5.0
Nurse/midwife ^∗∗∗^^or^^∗∗∗∗^	404	46.1
Laboratory technician ^∗∗∗^^or^^∗∗∗∗^	65	7.4
Physician assistant ^∗∗∗∗^	28	3.2
Pharmacist/dispensary officer ^∗∗^^or^^∗∗∗∗^	64	7.3
Health info. officer/biostatistician ^∗∗^^or^^∗∗∗∗^	75	8.6
Nutrition officer ^∗∗^^or^^∗∗∗∗^	40	4.6
Disease control officer ^∗∗^^or^^∗∗∗∗^	29	3.3
Other professions ^∗∗∗^^or^^∗∗∗∗^	128	14.6
*Respondent’s educational level*		
PhD	10	1.1
Master’s degree	80	9.1
Bachelor’s degree	389	44.4
HND/diploma	366	41.7
Certificate	18	2.1
SHS	11	1.3
Unspecified	3	0.3
*Respondent’s years of experience*		
0–5 years	470	53.6
6–10 years	232	26.5
11–15 years	103	11.7
16 years and above	72	8.2
*Mean = 6.77, SD* ^∗^ * = 6.021, range = 48*		

*Note:* Certificate = 2‐year professional certification at the tertiary level. The asterisks (∗) represent the number of years of education required. Source: survey of healthcare professionals who generate and use health information, 2023.

Abbreviations: HND, higher national diploma (both HND and diploma are 3‐year tertiary‐level education qualifications); SD, standard deviation; SHS, senior high school (3 years of secondary cycle education).

### 3.2. Scale Reliability of Research Constructs

The reliability of all the nine study constructs was determined using Cronbach alpha’s coefficient as a means of measuring and ensuring internal consistency. Following the study of Tavakol and Dennick [26], the study construct and its items had a tremendous measure for internal consistency as indicated in Table 3.

**Table 3:** Scale and item reliability test.

**Study construct**	**No. of items**	**Cronbach’s alpha**
Generic professional skills competency healthcare professionals	9	0.86
Competency in health information and records management	15	0.92
Competency in the use of language of healthcare	3	0.75
Competency in the use of healthcare terminologies and classifications	6	0.90
Competency in research methods skills	10	0.91
Competency in health service organization and delivery	6	0.85
Competency in health information law and ethics	4	0.80
Competency in electronic health skills	3	0.78
Competency in health information services organization and management	19	0.93
Overall tool’s reliability	75	0.97

### 3.3. The Overall CLoHCP Under All Nine Competency Areas of Focus

The results in Table 4 represent the overall competency levels recorded in each of the nine thematic areas of focus after the assessment. On a scale of 1–3, HCPs attained an advanced level competency category in three out of the nine areas of focus. HCPs were found to be of advanced competency in the application of generic professional skills (M = 2.33; CI: 95%, 2.30–2.37) and HI law and ethics (M = 2.50; CI: 95%, 2.38–2.53). In the remaining seven areas of competency, HCP attained an intermediate level based on the criteria of categorization used for this study: HI records and management skills (M = 2.27; CI: 95%, 2.13–2.32), use of language of healthcare (M = 2.00; CI: 95%, 1.93–2.12), healthcare terminologies and classification skills (M = 1.83; CI: 95%, 1.79–1.87), research methods skills (M = 1.94; CI: 95%, 1.91–1.98), health service organization and delivery skills (M = 1.96; CI: 95%, 1.91–1.99), electronic health skills (M = 2.06; CI: 95%, 2.02–2.10), and HI and service organization management (M = 2.00; CI: 95%, 1.96–2.03).

**Table 4:** The mean and proportion of competency level for all nine competency areas of focus.

**Indicator (competency area)**	**Competency level on a scale of**1–3 ± **S** **D**	**Competency level in percentage (%)**	**Competency level category**
Generic professional skills	2.33 ± 0.46	77.7%	Advanced
Health information records and management skills	2.27 ± 0.49	75.7%	Intermediate
Use of language of healthcare	2.00 ± 0.58	66.7%	Intermediate
Healthcare terminologies and classification skills	1.83 ± 0.59	61.0%	Intermediate
Research methods skills	1.94 ± 0.53	64.7%	Intermediate
Health service organization and delivery skills	1.96 ± 0.54	65.3%	Intermediate
Health information law and ethics	2.50 ± 0.54	83.3%	Advanced
Electronic health skills	2.06 ± 0.59	68.7%	Intermediate
Health information and service organization management	2.00 ± 0.49	66.7%	Intermediate

*Note:* Interpretations: 1.0–1.5 (entry level competency), 1.6–2.2 (intermediate level competency), and a targeted level of 2.3–3.0 (advance level competency). Competency level is presented as mean ± standard deviation and also in percentage with a target of 2.3/3.0. Source: survey of healthcare professionals who generate and use health information, 2023.

### 3.4. CLoHCP in All Nine Areas of Focus Categorized by Profession

Table 5 displays a cross‐tabulation of the CLoHCP involved in this study under each of the nine thematic areas of focus, which collectively form the overall competency level of the professionals. A glance at the table shows the competency level on a scale of 1–3 and its equivalent in percentage for each category of profession and thematic area. Under the generic professional skills column, all the categories of professions had a relatively high competency, with doctors, HI officers/biostatisticians, and physician assistants being the top three. Similarly, for the HI records and management skills, the competency level for the individual category of profession was quite impressive, with 2.5 and 2.0 being the maximum and minimum levels attained by HI officers/biostatisticians and nutrition officers, respectively.

**Table 5:** Mean and proportion of competency level of all nine thematic areas of focus categorized by profession type.

**Profession type**	**Generic professional skills**	**Health information records and management skills**	**Language of healthcare**	**Healthcare terminologies and classification skills**	**Research methods skills**	**Health service organization and delivery skills**	**Health information law and ethics**	**Electronic health skills**	**Health information and service organization management**
Doctor	2.6 (86.7%)	2.3 (76.6%)	2.3 (76.6%)	2.0 (66.7%)	2.2 (73.3%)	2.1 (70.0%)	2.5 (83.3%)	2.3 (76.6%)	2.2 (73.3%)
Nurse/midwife	2.3 (76.6%)	2.2 (73.3%)	2.2 (73.3%)	1.7 (56.7%)	1.9 (63.3%)	1.9 (63.3%)	2.4 (80.0%)	1.9 (63.3%)	1.9 (63.3%)
Laboratory technician	2.3 (76.6%)	2.2 (73.3%)	2.2 (73.3%)	1.8 (60.0%)	2.0 (66.7%)	2.0 (66.7%)	2.3 (76.6%)	2.1 (70.0%)	2.0 (66.7%)
Physician assistant	2.4 (80.0%)	2.2 (73.3%)	2.2 (73.3%)	2.0 (66.7%)	2.1 (70.0%)	2.0 (66.7%)	2.4 (80.0%)	2.2 (73.3%)	2.1 (70.0%)
Pharmacist/dispensary officer	2.4 (80.0%)	2.3 (76.6%)	2.4 (80.0%)	2.0 (66.7%)	2.1 (70.0%)	2.1 (70.0%)	2.4 (80.0%)	2.2 (73.3%)	2.2 (73.3%)
Health information officer/biostatistician	2.6 (86.7%)	2.5 (83.3%)	2.3 (76.6%)	2.1 (70.0%)	2.1 (70.0%)	2.2 (73.3%)	2.5 (83.3%)	2.4 (80.0%)	2.1 (70.0%)
Nutrition officer	2.2 (73.3%)	2.0 (66.7%)	2.0 (66.7%)	1.7 (56.7%)	1.9 (63.3%)	1.8 (60.0%)	2.2 (73.3%)	2.0 (66.7%)	2.0 (66.7%)
Disease control officer	2.4 (80.0%)	2.3 (76.6%)	2.2 (73.3%)	2.0 (66.7%)	2.1 (70.0%)	2.1 (70.0%)	2.4 (80.0%)	2.1 (70.0%)	2.0 (66.7%)
Other professions	2.3 (76.6%)	2.1 (70.0%)	2.1 (70.0%)	1.8 (60.0%)	1.9 (63.3%)	1.9 (63.3%)	2.4 (80.0%)	2.1 (70.0%)	2.00 (66.7%)

*Note:* Source: survey of healthcare professionals who generate and use health information, 2023.

Considering the competency in the use of the language of healthcare, the most competent category of the profession under this thematic area was found to be pharmacist/dispensary officer with a score of 2.4 and also HI officers/biostatisticians and doctors scoring close with a competency level of 2.3 each. Generally, the clusters of scores under that facet were considerably good for all HCPs. The findings reveal that HCP competence in healthcare terminologies and classifications is relatively low; this section saw most of the lowest scores. Only HI officers/biostatisticians had a score above 2.0, with four other professionals scoring exactly 2.0 (doctors, physician assistants, pharmacists/dispensary officers, and disease control officers). Professions like nurses/midwives scored as low as 1.7 out of 3.

On research methods and health service organization and delivery skills, six out of the nine categories of professionals scored 2.0 or more, except nurses/midwives, nutrition officers, and other professionals, scoring within a range of 1.8–1.9. Favorably, competency in HI law and ethics saw a recommendable competency rating: a maximum of 2.5 and a minimum of 2.2 for all the categories of professionals. Taking into cognizance the electronic health skills, all the professional types had a competency score of 2.0 or more, except that of nurses/midwives, scoring 1.9. The highest score, 2.4, emanated from HI officers/biostatisticians, followed by doctors with a score of 2.3. HI and service organization management saw many of the professions scoring within the range of 2.0 and 2.2 inclusively, and once again, only nurses/midwives scored 1.9, which was beneath the aforementioned range.

**Figure 1:**
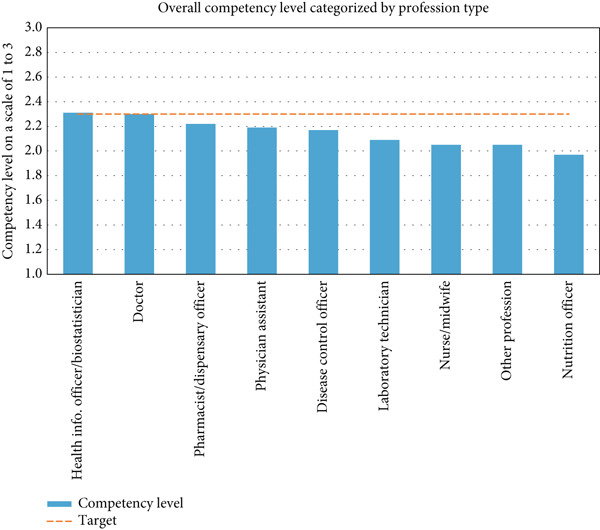
Overall competency level in the generation and usage of health information by healthcare professionals. *Source:* survey of healthcare professionals who generate and use health information, 2023.

Figure 1 shows the overall CLoHCP categorized by their professional type. The chart depicts that only the HI officers/biostatisticians and doctors attained competency ratings that were up to the targeted level of 2.3 or a percentage equivalent to 76.7%. Pharmacist/dispensary officers, physician assistants, and disease control officers had ratings very close to the target of approximately 2.2 each. The likes of laboratory technicians, nurse/midwives, nutrition officers, and other classifications of professionals had relatively low overall ratings for competency level.

### 3.5. Association Between Sociodemographic Characteristics of HCP and the CLoHCP for Generating and Using HI

In reference to Table 6, a bivariate chi‐square test of independence was conducted to ascertain and warrant a perfect conclusion for the study hypotheses as aforementioned in the methods of the study.

**Table 6:** Chi‐square test of independence between the various competency areas of focus and the sociodemographic characteristics of the healthcare professionals using a significance level of 5%.

**Indicators of competency**	**Sociodemographic characteristics**							
	**Sex**		**Profession**		**Educational level**		**Years of experience**	
	**χ** ^2^ **and** **p** **value** **(** **d** **f** = 4**)**	**Cramer’s** **V**	**χ** ^2^ **and** **p** **value** **(** **d** **f** = 16**)**	**Cramer’s** **V**	**χ** ^2^ **and** **p** **value** **(** **d** **f** = 12**)**	**Cramer’s** **V**	**χ** ^2^ **and** **p** **value** **(** **d** **f** = 6**)**	**Cramer’s** **V**
General professional skills	20.308, *p* = 0.001^∗∗^	0.108	32.544, *p* = 0.008^∗^	0.136	46.128, *p* = 0.001^∗∗^	0.162	72.034, *p* = 0.001^∗∗^	0.203
Health information records and management skills	11.406, *p* = 0.012^∗^	0.081	58.641, *p* = 0.001^∗∗^	0.183	32.636, *p* = 0.001^∗∗^	0.136	44.781, *p* = 0.001^∗∗^	0.160
Use of language of healthcare	7.968, *p* = 0.059	0.069	30.119, *p* = 0.017^∗^	0.131	49.424, *p* = 0.001^∗∗^	0.168	42.257, *p* = 0.001^∗∗^	0.156
Healthcare terminologies and classification skills	8.566, *p* = 0.036^∗^	0.074	49.988, *p* = 0.001	0.169	64.861, *p* = 0.001^∗∗^	0.192	26.974, *p* = 0.001^∗∗^	0.124
Research methods skills	18.794, *p* = 0.001^∗∗^	0.105	37.019, *p* = 0.002^∗^	0.145	69.551, *p* = 0.001^∗∗^	0.199	32.833, *p* = 0.001^∗∗^	0.137
Health service organization and delivery skills	8.884, *p* = 0.031^∗^	0.075	47.319, *p* = 0.001^∗∗^	0.164	56.106, *p* = 0.001^∗∗^	0.179	44.464, *p* = 0.001^∗∗^	0.159
Health information law and ethics	13.903, *p* = 0.004^∗^	0.088	32.663, *p* = 0.008^∗^	0.136	41.930, *p* = 0.001^∗∗^	0.155	34.599, *p* = 0.001^∗∗^	0.140
Electronic health skills	17.301, *p* = 0.001^∗∗^	0.108	73.688, *p* = 0.001^∗∗^	0.205	54.069, *p* = 0.001^∗∗^	0.176	17.944, 0.006 ^∗^	0.143
Health information and service organization management	11.531, *p* = 0.009^∗^	0.083	54.393, *p* = 0.001^∗∗^	0.176	80.171, *p* = 0.001^∗∗^	0.214	73.484, *p* = 0.001^∗∗^	0.205

*Note:* Source: author’s analysis of the survey of healthcare professionals who generate and use health information, 2023.

^∗^ *p* < 0.05 and  ^∗∗^ *p* < 0.001.

At a 5% significance level, the test was significant (*p* < 0.05) for almost all the pairs of variables as indicated in the table, except for “competency in the use of the language of health” and “sex.” The effect size of the significant association that existed between each pair of variables is indicated by Cramer’s *V*, as shown in the table. The strength of the associations ranged from 0.074 to 0.214, with the highest strengths of association being under the pairs which involved profession, educational level, and years of experience. Generally, the test result of our study is an indication that CLoHCP in all nine areas of focus is significantly associated with the sociodemographic characteristics of the HCPs.

### 3.6. Comparison of the Overall CLoHCP Recorded in All Eight Selected Health Facilities

The ANOVA test results displayed in Table 7 were significant (*p* < 0.001), indicating that there are significant differences in the overall competency levels of the professionals across the various healthcare facilities involved in the study. In addition to that, Bartlett’s test for homogeneity of variances was also significant (*p* = 0.002), warranting a conclusion that there are also differences in variances across the various facilities involved in the study. To unravel the exact pairs of facilities with different overall competency levels, the study employed a Dunnett’s T3 post hoc test conducted at a 5% significance level due to the heteroscedasticity of the various scores. As indicated in the table under Dunnett’s post hoc test, nine different pairs of health facilities out of the 28 possible pairs had significant differences in the overall mean scores of competency levels.

**Table 7:** One‐way ANOVA with a Dunnett’s T3 post hoc test at a 5% significance level for unravelling the differences in the overall competency levels among the healthcare facilities involved in the study.

**Descriptive statistics**			**Test of homogeneity of variances**		**ANOVA**	
**Facility**	**Mean**	**Standard deviation**	**Bartlett’s statistics**	**Sig.**	**F-statistic (** **n** = 877, **d** **f** = 7**)**	**Sig.**
UCCH	2.0165	0.36020	23.192	0.002	12.026	*p* < 0.001
SGH	1.9319	0.35335				
AMH	2.1863	0.45437				
EGH	2.0486	0.36854				
ENRH	2.3323	0.42401				
TGH	2.1890	0.30507				
AGH	2.0588	0.41199				
CCTH	2.0912	0.39765				
**Dunnett’s T3 post hoc test**						
**Facility**	**Mean differences**	**Sig.**	**95% confidence interval**			
			**Lower bound**	**Upper bound**		
SGH–AMG	−0.25441	0.001^a^	−0.4258	−0.0830		
SGH–ENRH	−0.40037	0.001^a^	−0.5595	−0.2412		
SGH–TGH	−0.25709	0.001^a^	−0.3919	−0.1223		
SGH–CCTH	−0.15935	0.030^a^	−0.3110	−0.0077		
EGH–ENRH	−0.28365	0.001^a^	−0.4518	−0.1155		
ENRH–AGH	0.27344	0.001^a^	0.0906	0.4563		
ENRH–CCTH	0.24103	0.001^a^	0.0723	0.4098		
ENRH–UCCH	0.31581	0.001^a^	0.1471	0.4845		
TGH–UCCH	0.17252	0.007^a^	0.0264	0.3187		

^a^Only pairs of facilities with significant differences have been presented on the table.

## 4. Discussion

### 4.1. Background Characteristics of Participants

We recorded a response rate of 97.7% emanating from more than six different categories of HCP in the selected healthcare facilities. The HCPs involved in the study were dominated by females (59.5%). On the professional level, many of the HCPs were nurses/midwives. The participants were mainly within the youthful age range (mean age = 33 years), having only 4.0% being 50 years or above. Many of the participants had attained a bachelor’s degree or HND/diploma as their highest level of education, with a few having master’s, PhD, secondary, and postsecondary certificates. More than half of the HCPs had years of experience of 1–5 years. Participants with 16 or more years of experience were 8.2% of the HCPs. However, the average years were approximately 7 years.

### 4.2. Generic Professional Skills Competency of HCP for Generating HI

The results of this study indicate that HCPs are very conversant with the general professional skills, which is an imperative prerequisite for proper generation and usage of HI during the discharge of their duties. The professionals had a high level of competency in all the nine dimensions used for measuring their overall generic professional skills competency; however, many were found to be more proficient in communication, teamwork, problem‐solving, and decision‐making, which concurs with what was put forward by [27], and also portray ethical behavior in the discharge of their duties. In addition to these, the study discovered that many of the HCPs had a better level of competency in the use of basic medical science and vocabulary and ICT which will in turn have a great impact on data generation and improve the delivery of care holistically [28]. Unequivocally, our study’s result serves as evidence buttressing the claim that HCPs have adequate competency levels when taking into cognizance general professional skills.

### 4.3. Specific Professional Skills Competency of HCP for Generating HI

Eight major domains were adapted and incorporated into measuring the specific competency level of the HCPs in the context of generating and using HI to increase the quality of care delivery [11]. The study probed and accessed all eight main domains to discover any peculiar lapses that existed. On account of competency in HI law and ethics of the HCP, our study saw a considerably high level (advanced) under the domain. The HCP had a marvelous level of skills and competency when it came to managing the health records of patients and adhering to ethical standards such as confidentiality, privacy, and disclosure of information amid the generation and usage of the information, coinciding with the findings of other scholarly works [29].

Notwithstanding the spectacular competency level of the HCP in HI law and ethics, the overall ratings for competency attained in the remaining seven domains were all within the intermediate levels, thus below the targeted competency level of 2.3 out of 3.0 (Table 4). In conformity with a study conducted by Nsiah et al., our study discovered that HCP competency in HI and records management was not up to standard [30]. During our study, we also discovered that many HCPs have very low competency, particularly in the use of healthcare terminologies and classification systems: coding morbidity and mortalities with the use of ICD‐10 and SNOMED CT to ensure quality reporting and usage of information. Specifically, nurses/midwives, medical laboratory technicians, and nutrition officers had relatively the lowest competency level in utilizing disease classification and coding systems. This could be a result of inadequate training of these particular professionals on these subjects. It is equally crucial for all HCPs to attain a basic level of knowledge of HI and its systems [31]. The findings solidify that the professionals have varying competencies in different domains [21]. Moreover, there was relatively low and absurd competency for research method skills and also that of healthcare service organization and delivery skills of HCP. The research design, statistical analysis, writing, and publishing skills of the professionals were not appealing. These lapses in competency can affect the quality of data generated, particularly its reliability, which will in turn have an awful effect on the usage of the data hence the need for immediate attention.

Furthermore, the findings of this study indicate that the HCP’s overall competency score on electronic health/digital health skills was great (Table 4). The findings of Shiferaw et al., in measuring the digital competency of HCPs, concluded that HCPs have low digital competency, but on the contrary, our study discovered that HCPs were proficient in the use of ICT to facilitate data management, ensuring data security and improving care delivery. However, on a professional categorization level, nurses and midwives had relatively low competency scores, unlike the results of other similar studies [29, 32]. The use of digital or electronic systems in the arena of HI has become pervasive and inescapable, and the utilization of these systems will only come to fruition when all genres of HCP harness their benefits [33].

When juxtaposing the overall competency levels by professional categorization, HI officers/biostatisticians and doctors were the only two categories of professionals that met the targeted level deployed for our study (Figure 1). This can be linked to how frequently these categories of professionals interact with data/information in the hospital, giving them more opportunities to gain much experience. The findings of the study suggest that HCPs who have less interaction with data tend to have low competency levels in the context of generating and using HI [29, 32].

### 4.4. Predictive Factors Associated With the Competency Levels of HCP for Generating HI

Unquestionably, factors such as the sociodemographic characteristics of the professionals can have implications on their level of competency as indicated in the works of many scholars [21, 34]. Our study deployed various statistical methods to ascertain if competency levels were affected by any sociodemographic characteristics. In Table 6, the results of our study imply that sex, profession type, educational level, and years of experience of HCP were all statistically significant predictive factors of competency levels as already established by other studies [22]. The educational level of a healthcare provider was a greater predictive factor or an influential factor of all the nine areas of competencies measured in our study. Proper education is an efficacious tool for inculcating the needed knowledge and skills for thriving in every sector of which the health sector cannot be left out [35]. In this vein, competency‐based education which has become an increasingly accepted blueprint for optimal success in all forms of education including health [36, 37] should be highly embraced. Not sideling the “years of experience,” it emerged as another major predictive factor which was highly associated with HCP’s competency. Competency in research methods skills, skills in healthcare delivery and organization, HI records and management skills, and HI and service organization management were all highly dependent on years of experience. Moreover, research method skills, electronic health skills, and generic professional skills of the HCP were also highly dependent on sex.

In furtherance, our study discovered via comparative statistical analysis as tabulated in Table 6 that there are significant differences in the overall competency levels in some pairs of the selected facilities. Nine of the 28 possible pairs of facilities had significant differences in overall competency levels for the generation and usage of HI. Facilities are situated in different jurisdictions and have also been occupied by different calibers of HCPs with diverse sociodemographic characteristics. These factors have already been proven to be predictive factors under this study and other renowned studies, and hence, it turns out to be only right to associate the differences unraveled in those pairs to it.

## 5. Conclusion and Recommendations

The study unveiled that HCPs have an adequate level of competency in basic generic professional skills, HI records and management skills, and skills in HI law and ethics, of which these play an integral role in the generation and usage of HI. Unexpectedly, there were lapses in some of the specific competency levels of HCP, notably, medical terminologies and disease classification skills, skills in research methods, and healthcare service delivery and organization skills. The levels of competency for generating and using HI varied on a professional basis, with nurses, midwives, medical laboratory technicians, and nutrition officers having the least unremarkable scores. Furthermore, the competency level of HCPs shows statistically significant dependency on their sex, professional type, educational level, and year of experience.

The following recommendations are suggested based on the findings garnered from this study:

• Conscious efforts toward increasing the importance of competency‐based education and other means of capacity building will be an effective panacea for curbing the competency gaps needed for quality HI and its usage.
• Moreover, we recommend proper supervision, in‐service training, collaborations, and teamwork for mitigating and eradicating issues in the competencies identified.
• Furthermore, it is recommended that workshops on ICD‐11 coding be organized for HCPs to improve their knowledge on how to document and report HI in conformity with international standards.
• Any interventional event organized should seek the indulgence of all categories of HCPs who partake in information generation and use it for other purposes.

## Conflicts of Interest

The authors declare no conflicts of interest.

## Author Contributions

R.O.B. contributed to the concept, design, data collection, analysis of the research, and manuscript writeup, and V.W.A., K.A.O.B., G.A., and N.K.M. contributed to the design, analysis, manuscript writeup, and review. E.E.A., A.I.N., G.S., S.E.B., W.D., I.A.S., and E.O. contributed to the data collection, manuscript writeup, and review.

## Funding

This study was self‐funded as part of academic work.

## Data Availability

The data that support the findings of this study are available in this article. The corresponding author may be contacted in case anyone needs the data.
